# No evidence for change in expression of *TBC1D1* and *TBC1D4* genes in cultured human adipocytes stimulated by myokines and adipokines

**DOI:** 10.1080/21623945.2021.1900497

**Published:** 2021-03-26

**Authors:** Łukasz Kępczyński, Szymon Wcisło, Irmina Korzeniewska-Dyl, Katarzyna Połatyńska, Agnieszka Gach, Dariusz Moczulski

**Affiliations:** a Department of Genetics, Polish Mothers’ Memorial Institute Research Hospital, Łódź, Poland; b Department of Internal Medicine and Nephrodiabetology, Medical University of Łódź and Military Medical Academy Memorial Teaching Hospital of the Medical University of Łódź - Central Veteran Hospital, Łódź, Poland; c Department of Thoracic, General and Oncological Surgery, Medical University of Łódź and Military Medical Academy Memorial Teaching Hospital of the Medical University of Łódź - Central Veteran Hospital, Łódź, Poland; d Department of Neurology, Polish Mothers’ Memorial Institute Research Hospital, Łódź, Poland

**Keywords:** TBC1D1, tbc1d4, cytokines, adipokines, irisin

## Abstract

TBC1D1 and TBC1D4 proteins play analogous, but not identical role in governing insulin-signalling pathway. Little is known about changes in expression levels of *TBC1D1* and *TBC1D4* genes in mammals, including humans. Number of factors were studied, but data remain controversial. The aim of this study was to evaluate the effect of selected cytokines, adipokines and myokines with known or putative insulin sensitivity regulation activity (adiponectin, irisin, omentin, interleukin 6, leptin, resistin, and tumour necrosis factor) on *TBC1D1* and *TBC1D4* expression levels in cultured differentiated human adipocytes. No significant differences were found between the adipocytes treated with different stimuli and this effect was determined not dose dependent. It is reasonable to conclude that relative shortage of data showing no change in TBC1D1 and TBC1D4 from literature results from publication bias; therefore, our finding provides additional insight into the role of both genes.

## Introduction

Two members of Tre-2/BUB2/CDC16 domain family – TBC1D1 (TBC1 domain family member 1) and TBC1D4 (TBC1 domain family member 4, also known as Akt substrate of 160 kDa, AS160) proteins play analogous, but not identical role in governing insulin-dependent docking and membrane fusion of glucose transporter GLUT4 containing vesicles, sequestered in intracellular compartment [1, 2]. TBC1D1 and TBC1D4 are key regulators of GLUT4 redistribution and serve as Rab GTPase activating proteins (Rab GAPs) [3], sharing Rab substrate specificity [4, 5]. Unphosphorylated, basal state TBC1D1 and TBC1D4 remain active, promoting their Rab substrates hydrolytic activity and keeping them in inactive (GDP-bound) forms, leading to GLUT4 sequestration. Phosphorylation, in response to glucose uptake stimuli, as insulin and muscle contraction, cause inhibition by phosphoprotein-binding protein 14-3-3 capture, and allows Rabs to become GTP-bound (active) and facilitate GLUT4 liberation to the plasma membrane. Recent data from murine model suggests co-operation between Tbc1d1 and Tbc1d4 in GLUT4 trafficking, with dominant role of Tbc1d1, and Tbc1d4 serving as precise modulator of insulin sensitivity [6].

TBC1D1 and TBC1D4 exhibit distinct abundance among insulin responsive tissues. Both proteins are highly, but variably, expressed in skeletal muscles [2,, 7]. Rodent Tbc1d4 protein is expressed in low levels in preadipocytes, but is induced during differentiation [8], whereas Tbc1d1 is present in murine adipocytes in much less abundance than its paralogue [9], and *Tbc1d1* gene shows different splicing pattern than in muscle cells [7]. Human subcutaneous adipose tissue exhibits opposite patterns of *TBC1D1* and *TBC1D4* expression than rodents – levels of *TBC1D1* mRNA was shown to be threefold greater than *TBC1D4* [10].

Little is known about changes in expression levels of *TBC1D1* and *TBC1D4* genes. Number of factors were studied, but data remains controversial. Diabetes was shown to down-regulate the expression of *TBC1D4* in skeletal muscle and adipose tissue of humans and rodents [11, 12, 13, 14]. *Tbc1d1* was shown to be up-regulated in diabetic rat muscle [15]. One study showed no significant change in both TBC1D1 and TBC1D4 protein abundance in diabetic patients [16]. To the best of our knowledge, the expression regulation of *TBC1D1* in mammalian adipose tissue was not studied before. Therefore, we aimed to investigate the effect of selected cytokines, adipokines and myokines with known or putative insulin sensitivity regulation activity (adiponectin, cleaved FNDC5/irisin, intelectin 1/omentin, interleukin 6, leptin, resistin, and tumour necrosis factor) on *TBC1D1* and *TBC1D4* expression levels in cultured differentiated human adipocytes.

## Material and methods

### Reagents

Hanks balanced salt solution (HBSS, 02–015-1A), penicillin-streptomycin-amphotericin B solution (03–033-1B), penicillin-streptomycin solution (03–031-1B), phosphate buffered saline (PBS, 02–020-1A), Red blood cells lysis buffer (RBCL, 01–888-1B), Dulbecco’s modified Eagle’s medium with high glucose (DMEM-HG, 01–052-1A), Ham’s F-12 supplement (01–095-1A), Foetal bovine serum (04–007-1A), Tripsin Solution (03–046-5B) and leptin (30-T683-A) were purchased in Biological Industries. Collagenase from clostridium difficile (C0130), 3-isobutyl-1-methylxanthine (IBMX, I5879), human insulin solution (I9278), dexamethasone (D4902), 3,3ʹ,5-triiodothyronine (T_3_, T6397), rosiglitazone (R2408), biotin (B4639), pantothenic acid (P5155), iron-saturated transferrin (T0665), adiponectin (SRP4901), cleaved FNDC5/Irisin (SRP6284), Intelectin 1/Omentin (SRP4560), Interleukin 6 (H7416), Resistin (SRP4560), tumour necrosis Factor (H8916) and Amplification grade DNse I (AMPD1) were purchased in Sigma Aldrich. iScript Advanced cDNA Synthesis Kit for RT-qPCR (172–5037)) and iTaq Universal SYBR Green Supermix (172–5121) were purchased in Bio-Rad.

### Tissue handling, primary cells isolation and culture

Abdominal subcutaneous tissue samples were obtained, with informed consent, from 5 non-obese, non-diabetic and cancer-free male adult subjects during elective abdominal surgery for cholelithiasis. None of the subjects presented active inflammatory process, assessed by normal blood C-reactive protein. The protocol was approved by local Ethics Review Board of Łódź Medical University number RNN/215/16/KE.

All tissue samples were transported to the cell culture unit in HBSS with antibiotics (penicillin, streptomycin and amphotericin B), and were subjects of immediate processing. All samples were mechanically minced and digested with 0.2% collagenase from Clostridium histolyticum in HBSS for 45 min in 37°C. The reaction was stopped by dilution 1:10 with PBS-EDTA and all mixtures were passed through 250 µm mesh, followed by filtering through 100 µm cell strainer and centrifugation. The pellets were resuspended in 1 ml of RBCL Buffer, diluted 1:10 with PBS and filtered through 70 µm cell strainer. Following second centrifugation, cell pellets were resuspended in 5 ml of DMEM-HG/Ham’s F-12 with 10% FBS, antibiotics (penicillin, streptomycin and amphotericin B; after the first day of initial culture, amphotericin B was removed), and plated on T-25 dishes, preincubated for 2 hours in culture medium. Cells were maintained in for 2 weeks, until the first passage. After reaching 80% confluence, all cell lines were cryopreserved in culture medium supplemented with 10% DMSO for further procedures.

### Adipogenic terminal differentiation and stimulation

Thawed cells were cultured in the same conditions as primary isolates. For adipogenic terminal differentiation, cells were seeded on T-25 dishes, media were replenished thrice a week. After reaching 80–90% confluence, adipogenic phenotype was induced in serum-free DMEM-HG/Ham’s F-12-based differentiation medium, containing 0.5 mM IBMX, 100 nM human insulin, 100 nM dexamethasone, 2 nM T_3_, 1 µM rosiglitazone, 33 µM biotin, 17 µM pantothenic acid and 10 µg/mL iron-saturated transferrin. Cells were maintained in differentiation medium until all visible cells acquired adipocyte morphology (this time was individually variable from 14 to 21 days). Adipogenic differentiation was assessed by visual inspection and confirmed by qPCR for *LEP* gene expression. After adipocyte phenotype induction, cells were either maintained in serum-free DMEM-HG/Ham’s F-12-based medium, supplemented with 10 nM human insulin, 10 nM dexamethasone and antibiotics for control samples or stimulated with chosen cytokine, myokine and adipokine for 24 h in the same maintenance medium. Cell lines, isolated from two individuals, replicated in two biological replicates, were used to determine most stable reference genes. Cell lines isolated from remaining three individuals, replicated in three biological replicates, were used to determine genes of interest (GOI) – *TBC1D1* and *TBC1D4* relative expression. For stable reference gene determination, stimulants (adiponectin, cleaved FNDC5/irisin, intelectin 1/omentin, interleukin 6, leptin, resistin and tumour necrosis factor) were used in one (highest) concentration – 100 ng/μL, for assessing GOI relative expression, every stimulant was used in three concentrations – 25 ng/μL, 50 ng/μL and 100 ng/μL, concurrently with own control cell culture.

### RNA purification, processing and qPCR

Total RNA from cultured cells was extracted using TRI Reagent. Purity and yield assessment was performed by spectrophotometric absorbance measurement at 230 nm, 260 nm and 280 nm. For qPCR analysis, RNA samples (1 to 3 µg) were digested with DNase I and reverse transcribed (iScript Advanced cDNA Synthesis Kit for RT-qPCR) according to manufacturer’s instructions. cDNA samples (2 μl RT each reaction product) were amplified in triplicates, along with No-Template and No-Reverse Transcription controls, using iTaq Universal SYBR Green Supermix, according to manufacturer’s recommendations on CFX96 Touch Platform (Bio-Rad). Primers, spanning exon-exon junction, were designed using free Primer-BLAST tool [17]. All primers used are listed with their sequence in Table 1. Five reference genes (*B2M, UBC, TFRC, SDHA*, and *HPRT1*) were tested to determine two most stably expressed in experimental conditions.

**Table 1.** Primer sequences

Gene	Forward primer	Reverse primer	Amplicon size (bp)
*TBC1D1*	CACCCAGTGCCACTCGATTT	TGGCTTTATTACCCCGGGAC	102
*TBC1D4*	CACCCACCTTCAAGCACAAAG	GAACTGACGAAAGATTGCTGC	118
*B2M*	AGCAGCATCATGGAGGTTTGA	AAAGCAAGCAAGCAGAATTTGG	70
*SDHA*	TCAGCATGCAGAAGTCAATGC	CGAACGTCTTCAGGTGCTTT	120
*UBC*	CAGCCGGGATTTGGGTCG	CACGAAGATCTGCATTGTCAAGT	72
*HPRT1*	CCCTGGCGTCGTGATTAGTG	CACCCTTTCCAAATCCTCAGC	91
*TFRC*	GCAAAATCCGGTGTAGGCAC	CTGGGCTGAAACCCATCTTT	86
*LEP*	ACATTTCACACACGCAGTC	TTGGATAAGGTCAGGATGGG	91

### Data analysis

Quantification cycles (C_q_) were calculated automatically by Bio-Rad CFX Manager Software v. 3.1. Both GOI were normalized to two most stably expressed genes in experimental condition (*B2M* and *SDHA*), as determined by *geNorm* algorithm [18]. Relative quantities were log_2_ transformed. The relation between relative expression and stimulant concentration was assessed using ANOVA (all samples) and Student *t* test for Pearson correlation coefficient *r* (to determine dose dependency). P-value below 0.05 was considered significant. Results were reported as fold change. Statistical analysis was carried out using R (The R Project for Statistical Computing, www.r-project.org).

## Results

**Figure 1.**
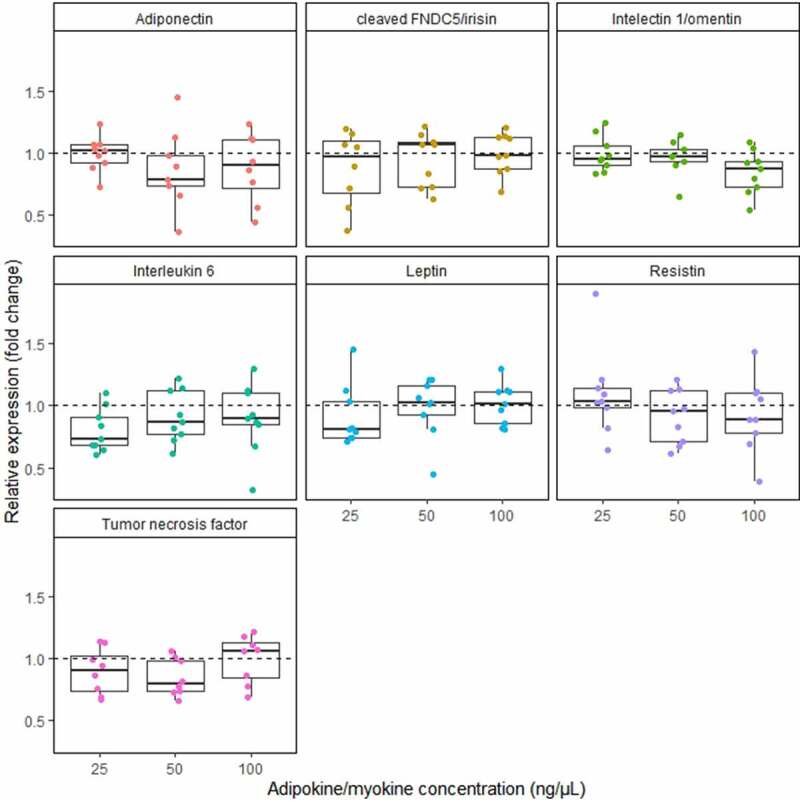
Effect of the increasing concentration of adiponectin, cleaved FNDC5 (irisin), intelectin 1 (omentin), interleukin 6, leptin, resistin, and tumour necrosis factor on *TBC1D1* gene expression on mRNA level. data are expressed as relative expression (fold change). stimuli concentration given in ng/μl. dashed line represents 1.0 relative expression (no change in expression between stimulated and control adipocytes)

**Figure 2.**
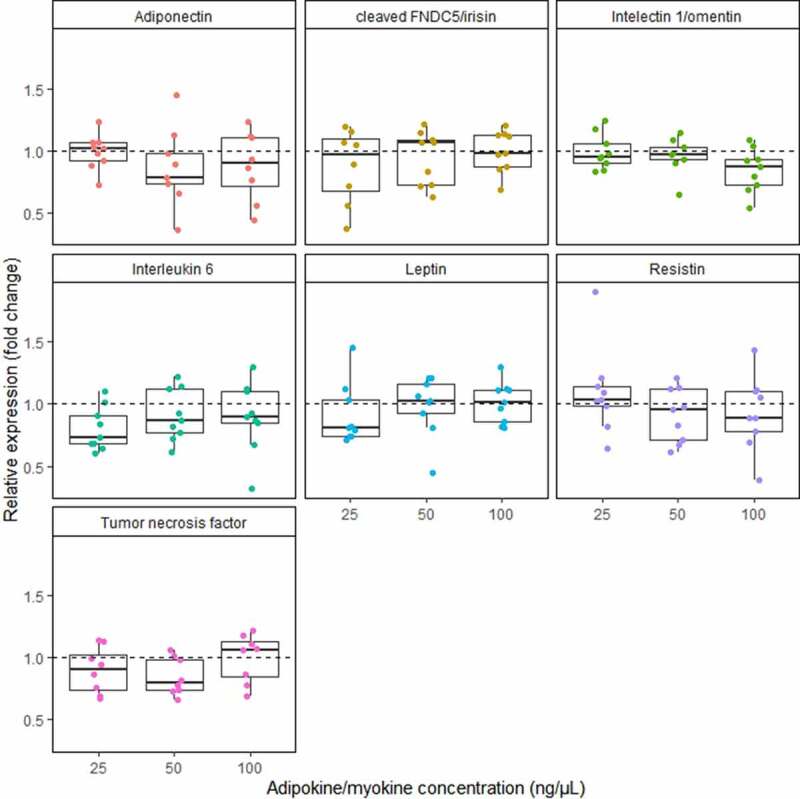
Effect of the increasing concentration of adiponectin, cleaved FNDC5 (irisin), intelectin 1 (omentin), interleukin 6, leptin, resistin, and tumour necrosis factor on *TBC1D4* gene expression on mRNA level. data are expressed as relative expression (fold change). stimuli concentration given in ng/μl. dashed line represents 1.0 relative expression (no change in expression between stimulated and control adipocytes)

Figures 1 and 2 present the effect of adiponectin, cleaved FNDC5 (irisin), intelectin 1 (omentin), interleukin 6, leptin, resistin and tumour necrosis factor on relative expression of *TBC1D1* gene (Figure 1) and *TBC1D4* gene (Figure 3). No significant differences were found between the cultures treated with different stimuli. We observed a high dispersion of expression change. All effects were determined as not dose dependent.

## Discussion

Skeletal muscles, immune cells and adipose tissue communicate through complex network of myokines, cytokines and adipokines to regulate organismal insulin sensitivity. Adipokines, as leptin, adiponectin, resistin and omentin/intelectin 1, produced in adipose tissue, may induce physiological response in effector cells outside adipose tissue, as well as in adipocyte tissue. Adipose tissue express leptin receptors, adiponectin receptors and bona fide resistin receptors; omentin/intelectin 1 receptor has not been identified so far. Cleaved FNDC5/irisin was identified as insulin sensitizer, secreted by exercising muscles; irisin receptor has not been identified so far as well. Both tumour necrosis factor and interleukin 6 are cytokines, known to be involved in inflammation-induced insulin resistance of adipose tissue. We investigated whether selected adipokines (adiponectin, leptin, resistin and omentin/intelectin 1), cytokines (tumour necrosis factor and interleukin 6) and myokines (cleaved FNDC5/irisin) of known or putative role in insulin sensitivity regulation, change the expression of genes for two key regulators of insulin signalling: *TBC1D1* and *TBC1D4*. We have observed no significant effect of adipokines and myokines on both genes expression in cultured human subcutaneous adipocytes.

Plethora of methods was used to determine the abundance of TBC1D1 and TBC1D4 on protein and mRNA level, including Western blotting [16, 19, 20, 21, 22, 23, 24, 25, 12, 26, 27, 28, 11, 29, 14], Reverse Transcription PCR (RT-PCR) [22], quantitative PCR (qPCR) [23, 20, 30, 31, 12, 26, 27, 32, 28, 33, 14], microarray [20] and RNA sequencing (RNA-seq) with qPCR validation [34, 13, 15].

TBC1D4 expression was studied on different mammalian models, including human [24, 16, 12, 34], mouse [20, 23, 26, 27, 29, 33], rat [22, 21, 28, 11, 13, 14], mares [19], and pigs [30]. Skeletal muscle was most extensively studied tissue, including muscle biopsy samples as well as cultured myocytes [16, 19, 20, 22, 21, 24, 30, 26, 27, 11, 28, 13, 33], followed by adipose tissue and cultured preadipocytes/adipocytes [16, 24, 30, 23, 12, 28, 29, 14]. Type 2 diabetes was shown to decrease expression of Tbc1d4 in skeletal muscle of streptozocin-treated rats [11] and diabetic Goto-Kakizaki rats (in comparison to control Wistar rats) [13]. In adipose tissue of females presenting diabetes and obesity the decrease in TBC1D4 expression in comparison to non-diabetic and non-obese subject was noted [12]. Babu et al. noted significant reduction of mRNA and protein level of Tbc1d4 in adipose tissue of diabetic rats [14]. Kampmann et al. showed no significant differences in TBC1D4 (and TBC1D1) expression in skeletal muscle among type 2 diabetic patients and matched controls [16]. Short-term high-sucrose diet significantly decreased Tbc1d4 protein expression in murine inguinal and retroperitoneal adipocytes [29], whereas high-fructose diet did not change the total amount of Tbc1d4 protein in murine skeletal muscle [26]. Tbc1d4 expression in murine skeletal muscle was significantly negatively correlated with fasting blood glucose concentration [33], whereas Kristensen et al. showed higher expression of TBC1D4 in high glucose presenting porcine adipose and pancreas but not muscle tissue [30]. Waller et al. did not find significant differences in TBC1D4 protein abundance among mares classified as insulin resistant and insulin sensitive [19]. Exercised rats muscle (soleus and epitrochlearis) did not show significant change in Tbc1d4 expression [21]. 17β-oestradiol enhanced the expression of *Tbc1d4* mRNA in ovariectomized rats gastrocnemius muscle, but not in mesenteric adipose tissue [28]. Murine Tbc1d4 expression was markedly down-regulated in skeletal muscles with age [27], TBC1D4 expression in human cumulus cells showed opposite relation [34]. Albers et al. showed significant up-regulation of TBC1D4 in adipose tissue and muscle tissue 12 months after gastric bypass surgery [24]. Co-culture of bacterial lipopolysaccharide stimulated macrophages with differentiated murine adipocytes reduced Tbc1d4 mRNA expression [23]. Aldosterone reduced the expression of Tbc1d4 rat muscle tissue [22]. Leptin reduced the expression of both Tbc1d1 and Tbc1d4 in skeletal muscle of ob/ob mouse [20].

Murine Tbc1d1 is expressed in adipocytes in lesser abundance than its paralogue Tbc1d4 [9]. Much less is known about changes in TBC1D1 expression in different physiological setups. Sáinz et al. described diminished expression of Tbc1d1 in ob/ob mice treated with leptin [20]. Tbc1d1 expression was significantly increased in diabetic Goto-Kakizaki rats muscle [15]. Preventive and therapeutic exercise group of rats (in comparison to sedentary group) exhibited increased Tbc1d1 expression in soleus and gastrocnemius muscles [32]. Myocardial infarct induces the expression of Tbc1d1 in murine heart muscle both in infarct zone and border zone [25]. Differences in Tbc1d1 expression in both adipose and muscle tissues were also noted between sexes in chicken model [31].

Expression changes of discussed genes remain controversial, and data from different studies might not be comparable due to differences in biological setups, including different stimuli as well as different material used (either biopsy specimen or cultured cells), interspecies variability and methodological issues. Being regulators of metabolism, both genes may be governed by different mechanisms in different species. It was shown that TBC1D1 variants are linked to obesity in many species, including human, rabbit and chicken, while deletion of murine Tbc1d1 suppressed obesity in ob/ob mice [35]. Kinase activation pattern, including Tbc1d1 phosphorylation in murine skeletal muscles, did not show the same pattern in human muscles [36].

Different adipose tissue depots exhibit different expressional pattern, this study was performed on in vitro differentiated subcutaneous adipocytes. Further work is needed to confirm the observation on different adipocytes populations. Nevertheless changes in TBC1D1 and TBC1D4 expression in human adipocytes were not subject of thorough studies. Changes in expression levels were rather noted in addition in studies of regulation of other genes, involved in insulin signalling pathway regulation. It is reasonable to conclude that relative shortage of data showing no change in TBC1D1 and TBC1D4 results from publication bias; therefore, our findings provide additional insight into the role of both genes.
